# Everyday Goal Pursuit in Older Couples: Lessons Learned From Electronic Daily Life Assessments

**DOI:** 10.1093/geroni/igaa057.1334

**Published:** 2020-12-16

**Authors:** Elizabeth Zambrano Garza, Christiane Hoppmann, Maureen Ashe, Denis Gerstorf, Kenneth Madden, Victoria Michalowski

**Affiliations:** 1 University of British Columbia, Vancouver, British Columbia, Canada; 2 Humboldt University Berlin, Berlin, Berlin, Germany; 3 University of British Columbia, Vancouver, Canada

## Abstract

Being able to progress on and accomplish personal goals is an important source of satisfaction and meaning across the adult lifespan and into old age. This study focuses on the importance of close others, such as spouses, for facilitating goal progress when individual resources may no longer suffice. We used multilevel modelling to analyze data from 119 couples (M age = 70 years, SD= 5.9; 50% women). Participants reported their personal goals at baseline and subsequently provided simultaneous ratings of goal pursuit, effort, and partner involvement three times daily over 7 days. Our findings show that more effort and spousal involvement indeed go hand in hand with better goal progress. More in-depth follow up analyses will pinpoint the role of individual and partner effects as potential mechanisms underlying contributions to everyday goal pursuit as well as address stress related processes.

